# A comparison of postoperative outcomes with PDA ligation in the OR versus the NICU: a retrospective cohort study on the risks of transport

**DOI:** 10.1186/s12871-018-0658-6

**Published:** 2018-12-22

**Authors:** Lisa K. Lee, Michelle Y. Woodfin, Marissa G. Vadi, Tristan R. Grogan, Phillip J. Ross, Richard L. Applegate, Marc Iravani

**Affiliations:** 1 Department of Anesthesiology and Perioperative Medicine, David Geffen School of Medicine, University of California, Los Angeles, 757 Westwood Plaza, Suite 3325, Los Angeles, CA 90095-7403 USA; 2 Department of Anesthesiology, Loma Linda University School of Medicine, Room 2532 LLUMC, 11234 Anderson Street, Loma Linda, CA 92354 USA; 3 Department of Anesthesiology and Pain Medicine , Section of Pediatric Anesthesiology, University of California at Davis School of Medicine, PSSB Ste 1200, 4150 V Street, Sacramento, CA 95817 USA; 4 Department of Medicine Statistics Core, David Geffen School of Medicine, University of California, Los Angeles, 911 Broxton Avenue, 3rd Floor, Los Angeles, CA 90024 USA

## Abstract

**Background:**

Although patent ductus arteriosus (PDA) ligations in the Neonatal Intensive Care Unit (NICU) have been an accepted practice, many are still performed in the Operating Room (OR). Whether avoiding transport leads to improved perioperative outcomes is unclear. Here we aimed to determine whether PDA ligations in the NICU corresponded to higher risk of surgical site infection or mortality and if transport was associated with worsened perioperative outcomes.

**Methods:**

We performed a retrospective cohort study of NICU patients, ≤37 weeks post-menstrual age, undergoing surgical PDA ligation in the NICU or OR. We excluded any infants undergoing device PDA closure. We measured the incidence of perioperative hypothermia, cardiac arrest, decreases in SpO2, hemodynamic instability and postoperative surgical site infection, sepsis and mortality.

**Results:**

Data was collected on 189 infants (100 OR, 89 NICU). After controlling for number of preoperative comorbidities, weight at time of procedure, procedure location and hospital in the mixed-effect model, no significant difference in mortality or sepsis was found (odds ratio 0.31, 95%CI 0.07, 1.30; *p* = 0.107, and odds ratio 0.40; 95%CI 0.14, 1.09; *p* = 0.072, respectively). There was an increased incidence of hemodynamic instability on transport postoperatively in the OR group (12.4% vs 2%, odds ratio 6.93; 95% CI 1.48, 35.52; *p* = 0.014).

**Conclusion:**

PDA ligations in the NICU were not associated with higher incidences of surgical site infection or mortality. There was an increased incidence of hemodynamic instability in the OR group on transport back to the NICU. Larger multicenter studies following long-term outcomes are needed to evaluate the safety of performing all PDA ligations in the NICU.

**Keywords:**

Patent ductus arteriosus, Newborn infant, Neonatal intensive care unit, Surgical wound infection, Postoperative period, Hemodynamics

## Introduction

The transport of extremely premature neonates from the Neonatal Intensive Care Unit (NICU) to the operating room (OR) is not without significant risk. These risks include inadequate monitoring, tracheal extubation, hypo/hyperventilation, loss of vascular access, discontinuation of life-sustaining infusions, acute hemodynamic deterioration and hypothermia. These risks are high in the immediate post-surgical period, when hemodynamic instability is more likely [1]. However, performing operative procedures in the NICU also presents risks. There may be increased difficulty in obtaining necessary surgical equipment and supplies, suboptimal lighting, or disruption of care to other neonates in the vicinity. There is also a theoretical increased risk of surgical site infection as bedside NICU procedures are completed outside the sterile confines of the operating room. However, premature neonates who had patent ductus arteriosus (PDA) ligation [2–5] and other bedside surgical procedures [1, 6–12] performed in the NICU have not been shown to have an increased incidence of surgical site infections thus far.

The feasibility, safety [2, 3] and cost-effectiveness [13] of performing PDA ligations, as well as other bedside NICU procedures were first addressed in the medical literature during the early 1980’s. This has since become an acceptable practice worldwide [4, 6, 12, 14]. Current practice varies across the United States. In some centers, all PDA ligations are performed in the OR, with only the most critically-ill patients being done in the NICU. In other centers, all PDA ligations are performed at bedside in the NICU. Additionally, some institutions send specialized teams to external sites to perform PDA ligations in order to eliminate the risks of transferring critically-ill neonates between institutions [15].

The question of whether it is safe or comparable to perform a PDA ligation at bedside in the NICU versus transporting the infant to the main OR has been previously evaluated [2–5], however, we believe this issue is worth revisiting as changes in medical practice have occurred. Advancements in the field of neonatology have allowed for the survival of smaller and more medically fragile premature infants [16]. Recently, it has been suggested that small changes in the homeostasis of physiologic parameters during their NICU stay may have profound effects on the neurodevelopment of these infants years later [17], making even more important to carefully assess the care provided to these patients. Here, we aim to examine the incidence of adverse outcomes associated with transport of critically-ill neonates between the NICU and the OR, and to also assess the risks of performing PDA ligations in the NICU in a comparison to those who have the procedure performed in the OR.

## Methods

After obtaining Institutional Review Board approvals, a retrospective chart review of all preterm NICU patients who had a surgical PDA ligation at UCLA Ronald Reagan Medical Center (RRUCLA) and UCLA Medical Center, Santa Monica (UCLASM) from January 2009 to January 2015 and at Loma Linda University Children’s Hospital (LLUCH) from February 2013 to April 2015 was performed. We chose to limit our study period to decrease the differences in outcome and survival of very low birthweight infants that were possibly related to improvements in neonatal care and should provide an accurate reflection of the outcomes of current practice.

Only preterm NICU infants up to 37 weeks post-menstrual age at the time of procedure were included. Those who had their PDA occluded in the cardiac catheterization lab were excluded from this study. Patients who had PDA ligation performed in addition to another procedure, such as central line placement, or as a part of an atrial septal defect, ventricle septal defect, or complex congenital heart repair were excluded. Patients were identified from billing records and cross-referenced with operating room schedules at the RRUCLA and UCLASM. At LLUCH, they were identified through a data query of the electronic medical record. Data was obtained from evaluation of NICU progress notes, surgical operative reports, anesthetic records, nursing flowsheets and discharge summaries.

At all three institutions, the main operating rooms are separated from the NICU by a considerable distance. During transport, all neonates remained in their isolettes and monitored in accordance to American Society of Anesthesiologist monitoring standards. Infants who were mechanically ventilated in the NICU were hand-ventilated during transport and peak airway pressures were monitored. The same vital sign monitoring system was used in the OR as in the NICU. The decision to have the PDA ligation performed in the NICU or to have the patient transported to the OR for the procedure was determined by the clinical judgement of the anesthesiologist, surgeon and neonatologist. Generally, infants who were smaller and more critically ill remained in the NICU for the procedure while older and less critically-ill patients were transported to the OR. At Ronald Reagan UCLA Medical Center and UCLA Medical Center Santa Monica, there was a preponderance of operating room procedures to NICU procedures, while at Loma Linda, this procedure was mostly performed in the NICU. For this reason, a mixed-effects model was employed for analysis.

For PDA ligations performed in the NICU, the infant remained in their isolette and was not moved to a specialized area. Temporary partitions were set up, and all personnel within the designated area were required to wear OR attire, hat and mask. All equipment and instruments to be used for the surgery were brought from the OR. Anesthesia was administered by pediatric anesthesiologists and the procedure was performed by pediatric general surgeons at UCLA and pediatric cardiac surgeons at LLUMC. In the OR group, a combination of low-dose volatile anesthetic and/or fentanyl infusion or bolus was administered. In the NICU group, fentanyl infusion or bolus was used, with the exception of one case where ketamine was also given. An intermediate-acting paralytic, such as rocuronium, was given in both groups. The infants were ventilated using the anesthesia machine in the OR, unless prematurity or severity of lung disease required the use of a neonatal ventilator. In the NICU, infants were maintained on the same neonatal ventilator.

The following baseline characteristic data were extracted from the chart of each patient: Sex, gestational age and post-menstrual age at the time of the procedure, birthweight, weight at the time of the procedure, type of ventilatory support, along with the number and type of comorbidities present at the time of the procedure. The use of and dosage of inotropes at time of procedure were also recorded. In order to standardize the measurement of inotropic support that each neonate required, we utilized a modified inotrope score [18]. The modified inotrope score was calculated as the sum of the inotrope dose, corrected for potency: (1 x dopamine [mg · kg^− 1^ · min^− 1^] + 1 x dobutamine [mg · kg^− 1^ · min^− 1^] + 100 x epinephrine [mg · kg^− 1^ · min^− 1^]). Intra-operative variables that were recorded included the location of the procedure and type of anesthetic used.

We recorded the incidence of perioperative hypothermia (defined as temperature ≤ 36.0 °C), intraoperative cardiac arrest requiring chest compressions, overall mortality prior to discharge, surgical site infection, and culture-confirmed postoperative sepsis. Decreases in SpO_2_ on arrival to the OR, as well as on arrival to the NICU were recorded. For patients who had PDA ligation performed in the NICU, these values were defined as the difference between the last recorded SpO_2_ prior to the start of the anesthetic record to the first recorded SpO_2_ for the procedure and the last SpO_2_ on the anesthetic record to the first recorded SpO_2_ by the NICU nurse after report was given, respectively. The incidence of hemodynamic instability, defined as greater than 20% change from baseline mean arterial pressure (MAP) in either direction on arrival to the OR, intraoperatively and on arrival back to the NICU, was also extracted from the patient records for the OR group. For the NICU group, these time periods were defined as the first blood pressure recorded after anesthesia start, lowest intraoperative blood pressure recorded and first blood pressure recorded after anesthesia stop time, respectively.

The incidence of transport-associated complications, such as tracheal extubation, loss of vascular access, and discontinuation of infusion medications, was recorded. Long-term outcomes that were investigated include total length of stay, length of stay after PDA ligation, number of days requiring ventilatory support after PDA ligation (defined as number of days between PDA ligation and successful extubation for greater than 3 days) and comorbidities arising after PDA ligation. We compared these variables and outcomes in infants who were transported to the main OR versus those infants who had their procedure performed in the NICU.

Statistical analyses were performed using SAS 9.4 (SAS institute, Cary, NC). Means and standard deviations were calculated for all continuous variables and frequencies were tabulated for categorical variables (stratified by OR/NICU). To assess which outcomes were associated with location, generalized linear mixed effects models were constructed. For continuous outcomes, linear mixed effects models were run including a term for NICU/OR and a hospital random effect which was collapsed into two categories, LLUCH and RRUCLA/UCLASM (as RRUCLA and UCLASM are staffed by the same physicians and practices between these locations are similar). Next, we ran the same set of models but included the additional fixed effects of weight at time of procedure and number of preoperative comorbidities. Finally, due to differing patient characteristics and conditions between the NICU and OR, we constructed a propensity score model to adjust for differences in birthweight, gestational age at birth, weight at time of procedure, age at time of procedure, number of preoperative comorbidities, need for inotrope, and inotrope score. Similar results were obtained in all three models. For dichotomous outcomes the same process was carried out using logistic mixed effects models. Residual analysis was performed to check model assumptions for normality/homoscedasticity. Six of the outcomes (birthweight, gestational age at time of birth, weight at time of procedure, corrected gestational age at time of procedure and inotrope score) were found to have potentially suspect residuals and a log transformation was performed for those variables. For continuous outcomes, average differences between OR and NICU groups were estimated from the models and presented with 95% confidence intervals (CI). For binary outcomes, the odds ratios between OR and NICU groups were estimated from the models and given with 95% confidence intervals. A *p*-value of ≤0.05 was considered statistically significant for all analyses.

## Results

### Preoperative variable analysis

One-hundred fifteen patients from RRUCLA and UCLASM and 74 cases from LLUCH met inclusion criteria for a total of 189 cases. Descriptive statistics from the cohort are shown in Table 1.

**Table 1:** Comparison of baseline patient demographic characteristics between operating room and NICU groups

	OR (*n* = 89)	NICU (*n* = 100)	Mixed Effect Models with Site Random Effect and Location		Mixed Effect Models with Site Random Effect, Location, Weight at Procedure and Number of Preoperative Comorbidities		Mixed Effect Model with Site Random Effect, Location and Propensity Score	
	Mean (SD)	Mean (SD)	Mean Difference (95% CI)	*p*-value	Mean Difference (95% CI)	*p*-value	Mean Difference (95% CI)	*p*-value
Birthweight (grams)^a^	929 (354)	771 (200)	0.23 (0.09, 0.36)	0.001	0.11 (−0.02, 0.23)	0.099	0.13 (0.01, 0.25)	0.032
Gestational Age at Birth (weeks)^a^	26.6 (3.0)	25.5 (2.0)	0.07 (0.03, 0.11)	< 0.001	0.03 (0.00, 0.07)	0.066	0.04 (0.00, 0.08)	0.029
Weight at Time of Procedure (grams)^a^	1252 (683)	864 (258)	0.30 (0.20, 0.41)	< 0.001	0.05 (0.01, 0.08)	0.016	0.10 (0.01, 0.19)	0.033
CGA at Time of Procedure (weeks)^a^	30.6 (4.4)	27.5 (2.2)	3.18 (2.21, 4.16)	< 0.001	0.92 (0.41, 1.42)	< 0.001	1.22 (0.37, 2.06)	0.005
Number of Preoperative Comorbidities	2.94 (1.5)	2.68 (1.3)	0.26 (−0.14, 0.67)	0.201	0.14 (−0.29–0.57)^b^	0.518^b^	0.07 (−0.37–0.52)	0.742
Inotrope Score^a^	2.8 (4.6)	4.9 (9.8)	−0.60 (− 1.06, − 0.14)	0.011	−0.46 (− 0.94, 0.01)	0.057	−0.43 (− 0.90, 0.05)	0.078
	OR (*n* = 89)	NICU (*n* = 100)	Mixed Effect Models with Site Random Effect and Location		Mixed Effect Models with Site Random Effect, Location, Weight at Procedure and Number of Preoperative Comorbidities		Mixed Effect Model with Site Random Effect, Location and Propensity Score	
	# (%)	# (%)	Odds Ratio (95% CI)	*p*-value	Odds Ratio (95% CI)	*p*-value	Odds Ratio (95% CI)	*p*-value
Number Requiring Inotropes (%)	34 (38%)	39 (39%)	0.73 (0.19, 2.82)	0.650	0.74 (0.18, 3.13)	0.683	0.63 (0.14, 2.80)	0.597
Number of Males (%)	51 (57.3%)	54 (54%)	1.09 (0.45, 2.63)	0.845	1.27 (0.51, 3.17)	0.613	1.58 (0.55, 4.57)	0.687
Ventilatory Requirements Prior to Procedure				0.001		0.005		0.006
Spontaneous	18 (20.2%)	1 (1%)	25.11 (3.23, 195.07)	0.002	10.38 (1.22, 88.32)	0.032	9.19 (1.09, 77.72)	0.042
NIMV	4 (4.5%)	4 (4%)	1.13 (0.27, 4.70)	0.866	1.10 (0.23, 5.17)	0.908	0.83 (0.17, 4.02)	0.811
Conventional	66 (74.2%)	46 (46%)	3.37 (1.81, 6.26)	< 0.001	5.53 (2.65, 11.51)	< 0.001	4.98 (2.38, 10.39)	< 0.001
HFOV/HFJV	1 (1.1%)	49 (49%)	0.01 (0.00, 0.09)	< 0.001	0.01 (0.00, 0.11)	< 0.001	0.02 (0.00, 0.12)	< 0.001

^a^log transformation of this variable was performed

^b^Number of preoperative comorbidities was omitted as a predictor from the model for this particular variable

Clinical data on the 189 premature infants who had PDA ligations performed in the operating room (OR) or the neonatal intensive care unit (NICU). For continuous variables, standard deviations are listed in parentheses. For categorical variables, the number of cases is given, followed by the percentage in parentheses. Abbreviations used: *CGA* corrected gestational age (estimated post-conceptual age weeks + post-natal age weeks), *NIMV* noninvasive mechanical ventilation, *HFOV* high frequency oscillatory ventilation, *HFJV* high frequency jet ventilation

**Fig. 1:**
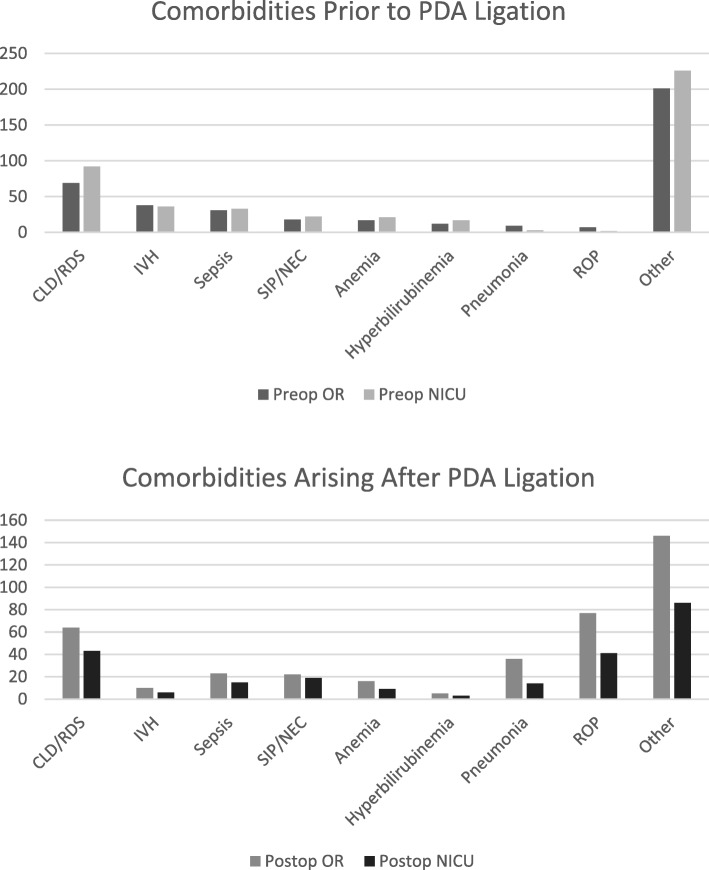
Histogram distribution of preoperative comorbidities and comorbidities arising after PDA ligation are shown. These were not included in the propensity scores or in final analysis due to insufficient numbers to perform a comparison

Analysis of the preoperative variables showed no significant difference in sex composition for either groups (*p* = 0.845). The mean number of preoperative comorbidities present also was not significantly different between the two groups (2.68 vs 2.94; *p* = 0.201). A distribution of the preoperative comorbidities and the number of comorbidities arising after PDA ligation are shown in Fig. 1. The NICU cohort had a statistically significant lower birthweight, weight at time of procedure, gestational age, and post-menstrual age at time of procedure, compared to the OR cohort. However, the proportion of those who were small for gestational age versus appropriate for gestational age at time of birth were similar between the OR (17 and 76%, respectively) and NICU groups (13 and 84%, respectively). The percentage of neonates on inotropic support at the time of procedure was not significantly different between the groups, after controlling for weight at time of procedure and number of preoperative comorbidities (odds ratio 0.73, 95% CI 0.19, 2.82; *p* = 0.650). Using a modified inotrope score to account for differences in type of inotrope and dosage [18], we found that the log of the mean inotrope score for the NICU group was higher than in the OR group, and that this was statistically significant (*p* = 0.011).

### Intraoperative variable and outcomes analysis

Of the PDA ligations that were performed in the OR, the majority of patients were given low-dose volatile agent in addition to opioid and paralytic (86 cases, 95%) with a few exceptions where only opioid and paralytic were given (4 cases, 4%). Maintenance of anesthesia in all PDA ligations performed in the NICU was accomplished with opioid and paralytic. A summary of intraoperative variables and outcomes are listed in Table 2.

**Table 2:** Comparison of intraoperative variables and outcomes in the operating room versus NICU groups

	OR (*n* = 89)	NICU (*n* = 100)	Mixed Effect Models with Site Random Effect and Location		Mixed Effect Models with Site Random Effect, Location, Weight at Procedure and Number of Preoperative Comorbidities		Mixed Effect Model with Site Random Effect, Location and Propensity Score	
	Mean (SD)	Mean (SD)	Mean Difference (95% CI)	*p*-value	Mean Difference (95% CI)	*p*-value	Mean Difference (95% CI)	*p*-value
Change in SpO2 from Baseline to Arrival in OR	0.58 (3.63)	−0.57 (4.76)	−1.15 (−2.39, 0.09)	0.069	−1.22 (−2.55, 0.10)	0.070	−0.95 (− 2.32, 0.42)	0.174
Change in SpO2 on Exit from OR to NICU	−1.47 (4.11)	−0.23 (3.14)	1.23 (0.19, 2.28)	0.021	1.03 (−0.09, 2.15)	0.070	1.37 (0.21, 2.53)	0.021
	OR (*n* = 89)	NICU (*n* = 100)	Mixed Effect Models with Site Random Effect and Location		Mixed Effect Models with Site Random Effect, Location, Weight at Procedure and Number of Preoperative Comorbidities		Mixed Effect Model with Site Random Effect, Location and Propensity Score	
	# (%)	# (%)	Odds Ratio (95% CI)	*p*-value	Odds Ratio (95% CI)	*p*-value	Odds Ratio (95% CI)	*p*-value
Intraoperative Hypothermia (# infants with temp ≤36 °C)	20 (26.3%)	5 (12.2%)	2.57 (0.88, 7.55)	0.085	2.25 (0.74, 6.89)	0.154	1.83 (0.57, 5.86)	0.306
Postoperative Hypothermia (# infants with temp ≤36 °C)	9 (12%)	5 (5.1%)	2.26 (0.70, 7.26)	0.172	1.54 (0.42, 5.59)	0.510	1.59 (0.43, 5.94)	0.489
Loss of Vascular Access	2 (2.2%)	1 (1%)	2.28 (0.20, 25.94)	0.506	1.72 (0.12, 24.29)	0.687	0.84 (0.06, 12.28)	0.895
Hemodynamic Instability on Arrival to OR	5 (5.6%)	3 (3%)	0.61 (0.12, 3.13)	0.552	1.05 (0.18, 6.33)	0.954	0.88 (0.16, 4.94)	0.880
Intraoperative Hemodynamic Instability	34 (38.2%)	42 (42%)	0.87 (0.48, 1.57)	0.642	0.75 (0.40, 1.41)	0.364	0.68 (0.35, 1.31)	0.243
Hemodynamic Instability on Arrival to NICU	11 (12.4%)	2 (2%)	6.93 (1.48, 32.52)	0.014	9.67 (1.95, 47.89)	0.006	5.88 (1.15, 30.01)	0.033

Intraoperative variables and outcomes of the 189 premature infants who had PDA ligations performed in the operating room (OR) or the neonatal intensive care unit (NICU). For continuous variables, standard deviations are listed in parentheses. For categorical data, the number of cases is given, followed by the percentage in parentheses. The percentage may not equal given number divided by n due to missing data for certain outcomes

Cases performed in the OR were found to be significantly longer than procedures done in the NICU. While some of this time can be accounted for in time taken to transport back to the NICU, the difference in length of case was greater than 20 min on average. To account for the uneven distribution of NICU cases that were performed at different hospitals and to also account for differences due to individual surgeon operating times, each location was analyzed separately for length of case. The average length of OR case at Loma Linda Medical Center was 12.97 (95% CI 8.63 to 17.31) minutes longer than in the NICU while the average length of OR case at UCLA was 35.92 (95% CI 23.84 to 48.00) minutes longer than in the NICU. Differences in length of cases performed in the OR were significant at both locations (*p* = 0.002 and *p* < 0.001, respectively). Furthermore, after accounting for the random hospital effect in our mixed effect model in addition to the fixed effect of OR/NICU, this difference remained statistically significant (*p* < 0.001), supporting the finding that, at a minimum, procedures done in the NICU do not take longer than those performed in the OR.

We found a statistically significant increased incidence of hemodynamic instability during the immediate postoperative period on transport back to the NICU for the OR group as compared to the NICU group (12.4% vs 2% respectively, odds ratio 6.93; 95% CI 1.48 to 35.52; *p* = 0.014). Statistical significance was still present after controlling for number of comorbidities present prior to PDA ligation and weight at time of procedure in our mixed effect model (*p* = 0.006). This also remained significant in the propensity score model, (*p* = 0.033).

There was a statistically significant decrease in SpO_2_ from time of exit from the OR to arrival in the NICU in the OR group (mean decrease of 1.47, SD = 4.11) compared to the NICU group (mean decrease of 0.23, SD = 3.14; difference − 1.15; 95% CI -2.39, 2.28; *p* = 0.021). However this decrease was very small and was not clinically relevant. In the mixed effect model adjusting for weight at time of procedure and number of preoperative comorbidities, this term did not reach statistical significance (*p* = 0.070), however the effect observed in the propensity score model parallels that of the random effects model (*p* = 0.021). There was also an increased incidence of hypothermia during the intraoperative and postoperative period in the OR group when compared to the NICU group (26.3% vs 12.2 and 12% vs 5.1% respectively), but this did not reach statistical significance in any of the three models.

Differences between the OR and NICU groups in other intraoperative outcomes, including incidence of cardiac arrest, unintended tracheal extubation, loss of vascular access or hemodynamic instability in the perioperative period did not reach statistical significance. It is interesting to note that there were three episodes of perioperative cardiac arrest that occurred in the OR group but none in the NICU group. In all instances, the arrest occurred either on arrival into the OR or shortly after induction. Although this did not reach statistical significance, this observation remains very concerning since infants who were selected to have their PDA ligations done in the operating room were deemed to be stable enough for transport.

### Postoperative outcomes analysis

A summary of the postoperative outcomes are listed in Table 3. The number of comorbidities arising after PDA ligation, days requiring ventilator support and length of stay after PDA ligation did not differ between the OR and NICU groups. Mortality and rates of sepsis were higher in the NICU group (14.3 and 62% respectively) than in the OR group (5.1 and 20%), consistent with preoperative variables that indicated this group had a higher morbidity burden compared to the OR group. After controlling for the number of preoperative comorbidities and weight at time of procedure in the mixed effect model, neither mortality prior to discharge nor the increased incidence of sepsis arising after PDA ligation in the NICU group was statistically significant (odds ratio 0.33, 95% CI 0.09 to 1.23; *p* = 0.098, and odds ratio 0.31; 95% CI 0.07 to 1.30; *p* = 0.107, respectively). The propensity score model showed similar results for the outcome of mortality (*p* = 0.116), and sepsis arising after the procedure in the NICU group (*p* = 0.079). None of the mortalities that occurred in this study were documented to be caused by complications from the PDA ligation. There also were no documented cases of culture-proven sepsis that occurred concomitantly with a surgical site infection. Interestingly, there was one occurrence of a surgical site infection in the OR group and none in the NICU group, although this was not statistically significant in either model.

**Table 3:** Comparison of postoperative variables and outcomes in the operating room versus NICU groups

	OR (*n* = 89)	NICU (*n* = 100)	Mixed Effect Models with Site Random Effect and Location		Mixed Effect Models with Site Random Effect, Location, Weight at Procedure and Number of Preoperative Comorbidities		Mixed Effect Model with Site Random Effect, Location and Propensity Score	
	Mean (SD)	Mean (SD)	Mean Difference (95% CI)	*p*-value	Mean Difference (95% CI)	*p*-value	Mean Difference (95% CI)	*p*-value
# Comorbidities Arising after PDA Ligation	1.81 (1.75)	2.36 (1.42)	−0.05 (−0.75, 0.64)	0.876	0.20 (−0.49, 0.89)	0.566	0.23 (− 0.48, 0.94)	0.527
Days Requiring Ventilator Support^b^	22.20 (31.91)	22.30 (20.13)	−0.26 (− 0.58, 0.07)	0.121	0.04 (− 0.28, 0.36)	0.793	0.13 (− 0.22, 0.47)	0.473
Length of Stay after PDA ligation (days)	81.60 (48.86)	94.09 (28.21)	−12.49 (−27.45, 2.48)	0.101	−5.19 (−20.82, 10.44)	0.521	−4.34 (−20.30, 11.61)	0.591
	OR (*n* = 89)	NICU (*n* = 100)	Mixed Effect Models with Site Random Effect and Location		Mixed Effect Models with Site Random Effect, Location, Weight at Procedure and Number of Preoperative Comorbidities		Mixed Effect Model with Site Random Effect, Location and Propensity Score	
	# (%)	# (%)	Odds Ratio (95% CI)	*p*-value	Odds Ratio (95% CI)	*p*-value	Odds Ratio (95% CI)	*p*-value
Mortality^a^	3/59 (5.1%)	12/84 (14.3%)	0.33 (0.09, 1.23)	0.098	0.31 (0.07, 1.30)	0.107	0.32 (0.08, 1.33)	0.116
Sepsis Arising after PDA Ligation	18 (20%)	62 (62%)	0.36 (0.14, 0.90)	0.029	0.40 (0.14, 1.09)	0.072	0.42 (0.16, 1.11)	0.079

Postoperative outcomes in premature infants who had a PDA ligation performed in the neonatal intensive care unit (NICU) vs the operating room (OR). The number of cases is given, followed by the percentage in parentheses

^a^The mortality rate does not include patients who were transferred back to referring hospitals and lost to follow up, since it was unknown whether they survived to discharge to home. The denominator reflects the number of infants for whom there was follow-up documentation

^b^Log Transformation of this variable was performed

## Discussion

**Fig. 2:**
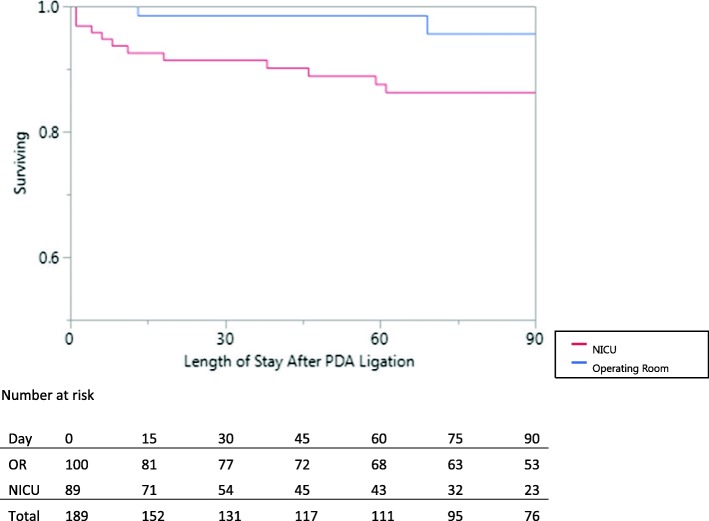
Unadjusted survival plot of infants who had PDA ligations performed in the NICU. No further mortalities were documented beyond 70 days in either group. All-cause mortality has decreased dramatically since the 1980’s when the mortality rate at 1 month post-procedure was quoted as 18–26% in premature infants who had their PDA ligations in the OR [2], and overall mortality of 38% in neonates who had PDA ligations done in the NICU

In this study, we did not find evidence for increased risk of surgical site infections associated with performing PDA ligations in the NICU, similar to the results of a study conducted by Gavilanes et al.) [10]. There was a higher mortality and sepsis rate in the NICU group from our linear mixed effect model, but this was not statistically significant after controlling for weight at time of procedure and number of preoperative comorbidities (*p* = 0.107 and *p* = 0.072, respectively). There were no mortalities that occurred secondary to the PDA ligations in the NICU group, which is also consistent with findings from previous studies [2, 3, 5, 19]. However, we did find that the all-cause postoperative mortality rate in the OR group (5.1%) and NICU group (14.3%) have fallen dramatically since the 1980’s (Fig. 2) when the mortality rate at 1 month post-procedure was quoted as 18–26% in premature infants who had their PDA ligations in the OR [2], and overall mortality of 38% in neonates who had PDA ligations done in the NICU [3].

For PDA ligations performed in the OR, we did find evidence of increased risk to the preterm infant, in contrast to an earlier study suggesting no difference in blood pressure associated with performance of the surgery in the OR versus NICU [8]. There was a statistically significant increased incidence of hemodynamic instability in critically-ill neonates as they were being transported back from the OR to the NICU in all 3 models (*p* = 0.014, *p* = 0.006, *p* = 0.033). This finding is particularly concerning since the OR group had greater post-menstrual age than the NICU group and were presumed to be robust enough to tolerate transport. Although it is possible that intraoperative sevoflurane administration could contribute to this finding, there was no increased incidence of intraoperative hemodynamic instability in the OR group, thus it is unlikely that the use of a volatile anesthetic was the sole etiologic factor contributing to the increased incidence of postoperative hemodynamic instability.

We chose to define hemodynamic instability as mean blood pressure change greater than or less than 20% of the patient’s baseline blood pressure. While this is an accepted definition [20], there are other accepted definitions of hypotension such as MAP below gestational age in weeks and MAP less than 10th percentile for gestational age/birthweight and postnatal age [21]. However, the latter definition is problematic due to the paucity of data defining what a normal blood pressure is in a preterm, very low birthweight but otherwise healthy infant beyond the first 5–7 days of life who is free from conditions that could affect their blood pressure [22]. The former definition is also not quite optimal as it was defined for awake infants [20] not under anesthesia, leading to a possible overestimation of the true incidence of clinically significant hypotension that ultimately results in poor outcomes.

The long-term significance of these alterations in hemodynamics is not immediately clear. It has been suggested that short periods of hemodynamic instability can lead to poor neurodevelopmental outcomes [17, 20, 23] and increase morbidity and mortality [16, 24]. In light of this, along with the known increased risk of mortality with hypothermia [16] and the lack of evidence for increased incidence of surgical site infection of PDA ligations performed in the NICU, consideration should be given to creating space for an OR in or close by the NICU to mitigate the risks of transport when planning future hospitals. A strong limitation of this study was the inability to specify the length of hemodynamic instability that would lead to poorer patient outcomes due to the retrospective data set and EMR factors. Such a mean BP target would need to be defined for each patient based on gestational age. Our study suggests a prospective study using automated data capture may identify a specific measure of hemodynamic instability (mmHg-minutes below a target) that correlates to worse outcome.

While we believe that our findings can be generalizable to other procedures performed in the NICU, we chose to analyze only one procedure to minimize the confounding effects of the type of surgery on clinical outcomes that were examined. The results we observed in this study suggest that the risks of transporting critically-ill neonates are real, however the long-term effects on neurodevelopmental outcome are still undefined. Further studies with larger cohorts are needed to delineate these risks and to evaluate the relationship between preoperative comorbidity, intraoperative events and postoperative outcomes.

Strengths of this study include the larger patient population compared to previous studies and the use of propensity scoring as a method to control for other factors besides procedure location that could have contributed to worsened outcomes. Despite this relatively larger sample size, the incidence of sepsis and death related to PDA ligation was extremely low to non-existent. Therefore, interpretation of this as no difference between the NICU and OR groups requires caution due to sparse data. Another limitation of this study is the inability to randomly assign treatment arms as this was a historical cohort study, however for ethical reasons, a randomized prospective study in the future is unlikely. There is also the possibility of underestimation of true morbidity and mortality postoperatively due to loss of follow-up in patients who were transferred back to their referring hospitals. We did note that the study period between the study sites differed, but overlapped. We do not believe that there was a drastic change in medical practice over that period of time that would change the results of our findings.

## Conclusion

There was no evidence for increased incidence of surgical site infection in the NICU group and after controlling for number of preoperative co-morbidities and weight at time of procedure, the increased incidence of sepsis arising after PDA ligation in the NICU group was not statistically significant. However, there was a statistically significant increased incidence of hemodynamic instability during transport back to the NICU in the OR group. Although this is a three-site study, larger multicenter studies with careful prospective data collection may identify a specific measure of hemodynamic instability (such as the number of minutes below a target) that correlates with a worse outcome are needed in order to evaluate the safety and efficacy of performing all PDA ligations in the NICU setting.

## Abbreviations

CI: Confidence Interval
LLUCH: Loma Linda University Children’s Hospital
MAP: Mean Arterial Pressure
NICU: Neonatal Intensive Care Unit
OR: Operating Room
PDA: Patent Ductus Arteriosus
RRUCLA: UCLA Ronald Reagan Medical Center
UCLASM: UCLA Medical Center, Santa Monica
